# Intestinal ulcers as an initial finding in EBV-associated lymphoproliferative disorder: A case report

**DOI:** 10.1097/MD.0000000000018764

**Published:** 2020-01-17

**Authors:** Sizhu Wang, Yinghuan Dai, Jie Zhang, Dalian Ou, Chunhui Ouyang, Fanggen Lu

**Affiliations:** a Department of Gastroenterology; b Department of Pathology, The Second Xiangya Hospital of Central South University, Changsha, Hunan, PR China.

**Keywords:** Crohn disease, Epstein–Barr virus, lymphoproliferative disease

## Abstract

**Rationale:**

Epstein-Barr virus (EBV)-associated T-cell lymphoproliferative disorder (LPD) usually occurs in children and young adults. Gastrointestinal involvement is rare. EBV-associated T-cell lymphoproliferative disorder manifesting as intestinal ulcers poses diagnostic challenges clinically and pathologically because of the atypical manifestations. We concluded that some indicators according to our case and literatures, which might be helpful to the diagnosis of EBV-associated LPD manifested as intestinal ulcers.

**Patient concerns:**

Here we present a 26-year-old man with complaints of diarrhea and abdominal pain that had persisted for 1 year. Multiform and multifocal deep ulcers were discovered in the colonoscopy. Cell atypia was not obvious but colitis with crypt distortion was found in pathology.

**Diagnoses:**

According to the symptoms, laboratory examinations, colonoscopy and pathology results, Crohn Disease was diagnosed.

**Interventions:**

Infliximab therapy was initiated based on the diagnosis of Crohn Disease.

**Outcomes:**

After the fifth course of therapy, intermittent fever and hematochezia occurred. Physical examination revealed swollen tonsils and ulcers, and purulent exudate from the right tonsil and palatoglossal arch were observed. Biopsies obtained through colonoscopy and nasopharyngoscopy demonstrated EBV-associated T-cell proliferation disease (level 3). After that, the tissue sample from the first colonoscopy was reexamined immunohistochemically. The result suggested EBV-associated T-cell proliferation disease (level 1).

**Lessons:**

When we confront with patients with multiform and multifocal deep intestinal ulcers, not only the common diseases such as Crohn Disease and intestinal tuberculosis should be considered, EBV-associated T-cell proliferation disease should be considered as well. Repeated multiple biopsy, gene rearrangement, EBV DNA quantitative analysis result, EBV-encoded RNA(EBER) and experienced pathologists might be helpful to the diagnosis.

## 1 Introduction

Epstein-Barr virus (EBV)-associated lymphoproliferative disease (LPD) is defined as EBV + B-cell LPD or EBV + T/NK-cell LPD.^[1]^ EBV-associated T-cell LPD is a category that was newly adopted by the World Health Organization (WHO) in 2008; its features include excessive lymphoid proliferation of T cells. It usually occurs in children and young adults.^[2,3]^ Gastrointestinal involvement is rare, and only several prior cases in immunocompetent hosts have been reported.^[4–6]^ EBV can be detected in > 95% of the human population in a chronically infected state (IgG positive).^[7]^ However, the long latency and reactivation of EBV results in various lymphoproliferative lesions, including hematological malignancies.^[8]^ Several cases of misdiagnosed EBV-associated LPD have been reported, but no typical manifestations were found. It poses diagnostic challenges clinically and pathologically. In this article, we present an uncommon case of EBV-associated T-cell LPD manifesting as intestinal ulcers. The immunocompetent patient was originally misdiagnosed as having Crohn disease and treated accordingly. This case could provide clinicians critical insight into the manifestation and treatment of this rare condition.

## 2 Case report

**Figure 1:**
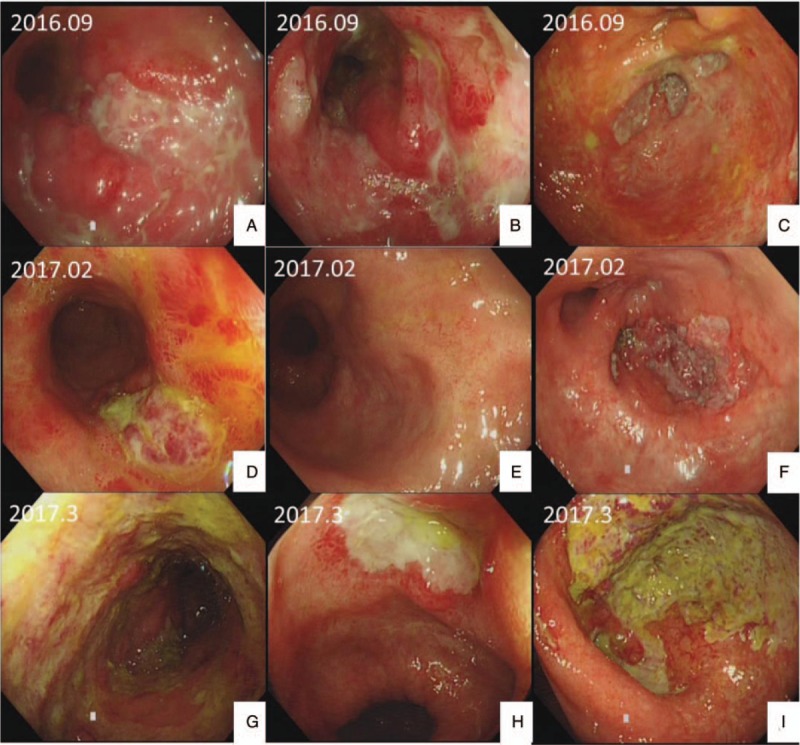
Endoscopic features. (A–C) 2016.09. A, Deep longitudinal ulcer in descending colon. Formation of mucosal island. B, Huge irregular shaped ulcer in transverse colon. C, Deep ulcer in cecum. (D–F)2017.02 Colonscopy revealed part of the ulcers were smaller or turned into scars. D, Ulcer in descending colon was smaller. E, Ulcers in transverse colon were repaired and turned into scars. F, Deep ulcer in cecum. (G–I) 2017.03 Colonscopy revealed mutiple deep huge ulcers. G. Huge circumferential ulcer in descending colon. H, Deep huge ulcer in transverse colon. I. Deep huge ucer in cecum.

**Figure 2:**
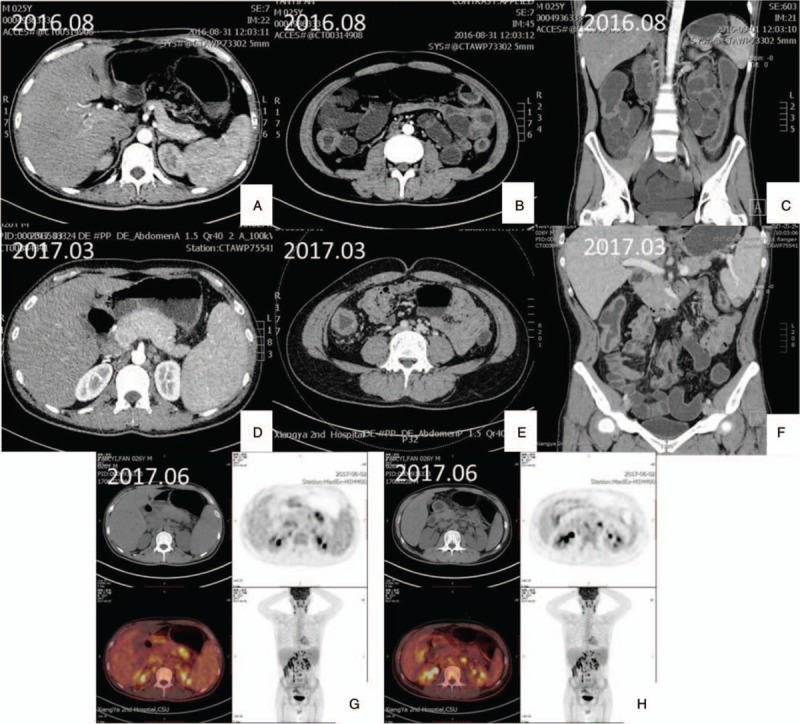
Intestine computed tomography and PET-CT. (A–C) 2016.08 An intestine computed tomography shows segmental wall of the cecum and descending colon thickened and intensive with multiple lymph nodes seen in the mesentery and retrperitoneal area. The diameter of the biggest one was 10 mm. The size of the spleen was a little bigger. (D–F) 2017.03 An intestine computed tomography shows more and bigger lymph nodes in the mesentery and retrperitoneal area with no obvious change in the colon. Liver, pancreas and spleen were bigger. There were mutiple small infarctions in spleen. (G–H) 2017.06 PET-CT shows there was a malignant tumor in the left nasopharynx wall, which may be lymphoma. Cervical and abdominal lymph nodes were swollen. Active glucose metabolism was detected in the swollen liver, pancreas and the lump in the tail of pancreas.

**Figure 3:**
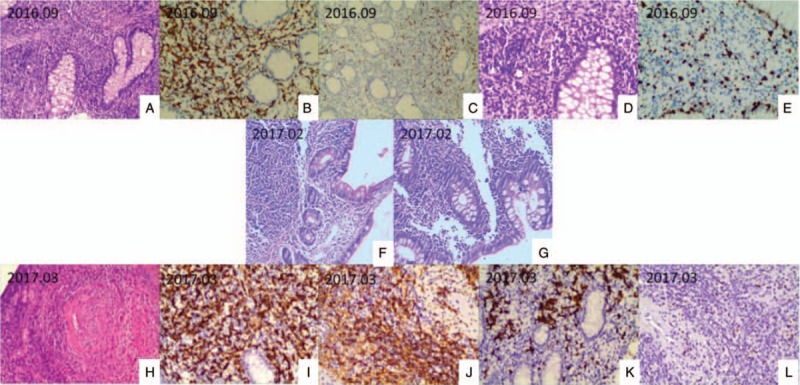
Histologic findings of the intestinal tissue. (A–E) 2016.09 Histologic findings of the cecum tissue under the microscope. A, Lymphocytic, plasma cell and eosnophis infiltration. B, The cells express CD3 (magnification, 200 × ). C, Part of the cells express CD20. D, Lymphocytic infiltration (magnification, 200 ×). E. The cells express KI67 scatteredly (magnification, 200 ×). (F-G) 2017.02 Histologic findings of the ascending colon and terminal ileum tissue under the microscope. Chronic mucositis and erosion with lots of lymphocytes and neutrophils infiltrating could be seen in the ascending colon. In the terminal ileum, chronic mucositis could be seen. The mesenchyme was loose and edema. Lots of lymphocytes infiltrated part of the area. No granuloma formation or tissue was discovered in both areas. (H-L) 2017.03 Histologic findings of the intestinal tissue under the microscope. H, Lymphocytes infiltrate vascular wall. I, The cells express CD3. J, The cells of the vascular wall express CD4. K, The cells express CD20. L, The cells of the vascular wall express EBER (magnification, H in 100 × and others in 200 ×).

A 26-year-old man was admitted to our hospital with complaints of diarrhea and abdominal pain that had persisted for 1 year. He never had fever and night sweats. His weight decreased by 10 kg since the onset of the symptoms. He had no alcohol consumption, drug abuse, relevant family history, or history of other severe diseases. His body temperature and blood pressure was normal. Physical examination revealed no positive sign. The laboratory test results were as follows: white blood count, 6.42 × 10^9^/L; red blood count, 4.58 × 10^12^/L; hemoglobin level, 121 g/L; and platelet count, 365 × 10^9^/L. In the routine stool examination, 1 + white cells were found but no red blood cells. OB was positive. The erythrocyte sedimentation rate was elevated to 29 mm/h, and the C-reactive protein level was high at 69.26 mg/L. Serum total protein and albumin levels were low at 46.6 g/L and 22.1 g/L, respectively. His liver and renal function test results and coagulant activity were normal. Negative results were obtained in T-SPOT test and serum virus laboratory tests (for cytomegalovirus CMV-IgM, CMV-IgG, EBV-IgM, and EBV-IgG). Computed tomography enterography (CTE) revealed thickening of the intestinal canal in portions of the descending colon and cecum, where enhancement was also observed. Multiple lymph nodes were found in the mesenteric and retroperitoneal areas (Fig. 2). The lung CT image showed no abnormal signs. Colonoscopy performed on September 9, 2016, revealed extensive deep ulcers with many morphological features, multiple nodular hyperplasia, and multiple deep ulcers from the ascending to the sigmoid colon. The ileocecal valve was edematous and distorted. Part of the intestinal canal was narrow (Fig. 1). Colonoscopic biopsy showed colitis with crypt distortion and infiltration of lymphocytes, neutrophils, and eosinophils in the lamina propria, but no evidence of enterophthisis. The immunohistochemical results were CK(−), CD3(+), CD20(+), CD21(follicular net+), and Ki-67(15%+) (Fig. 3). Considering synthetically the segmental ulcers and pebbly appearance, Crohn disease was suspected (Diagnostic criteria of Crohn disease was presented in Table 1). Crohn disease was an exclusive disease. According to the serological examination, infection of tuberculosis, CMV and EBV were excluded. Therefore, the patient was more likely suffering from Crohn disease and he was treated with infliximab therapy. Anti-tumor necrosis factor (TNF) therapy (infliximab) was administered 5 times, with good response. Colonoscopy performed in February 2017 revealed multiple ulcers. However, some ulcers were healed. Formation of white scars was observed in the ascending, transverse, and descending colon (Fig. 1).

**Table 1:**
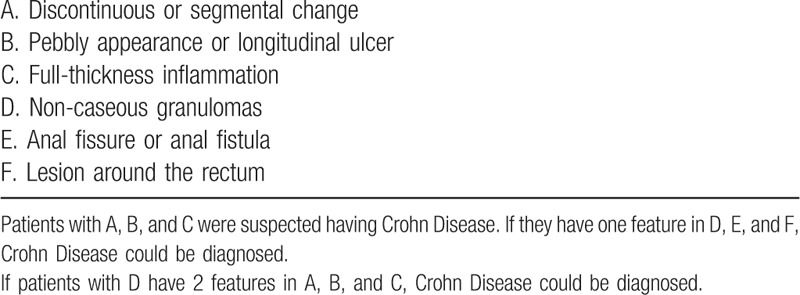
Diagnostic criteria of WHO for Crohn Disease.

**Figure 4:**
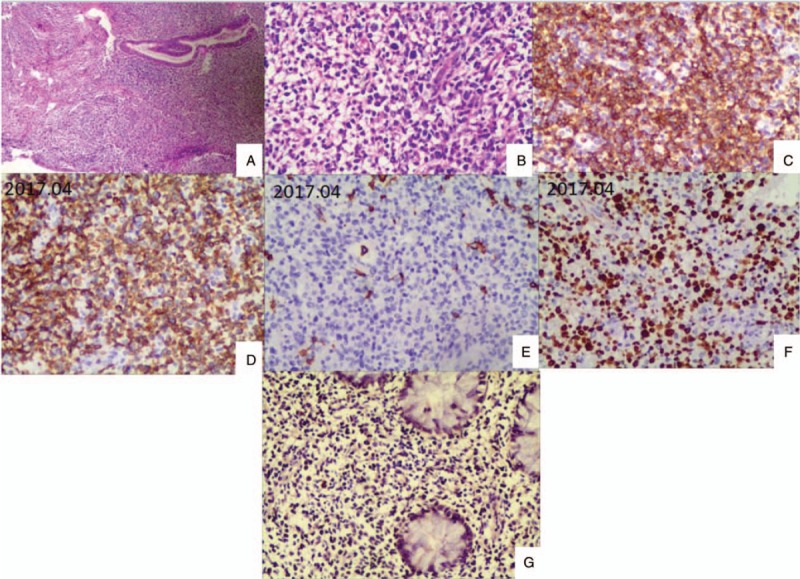
Histologic findings of the nasopharynx tissue and the cecum tissue got in the first colonscopy. (A–F) 2017.04 Histologic findings of the nasopharynx tissue under the microscope. A, Reduced glands. Lymphocytic infiltration. B, Atypical lymphocyte. Lots of fragmented remains. Immnohistochemistry results of CD2 expression (C), CD3 expression (D), CD20 focal expression(E), Ki-67 80% expression(F). G. Read the pathology of the cecum tissue got in the first colonscopy again. EBV related T cell proliferation disease (level 1, proliferation disease) was indicated. Immnohistochemistry results of EBER expression. (magnification, H in 100 × and others in 200 ×).

After almost 8 months——the fifth course of the anti-TNF therapy, the patient presented with intermittent fever (peak, 39.5°C) and hematochezia, and was readmitted to our department again. Furthermore, he coughed up white parenchyma and complained of sore throat. His body temperature was 38.1°C and blood pressure was normal. A physical examination revealed swelling of the tonsils and ulcers, and purulent exudate from the right tonsil and palatoglossal arch. On abdominal physical examination, we found no new positive sign but found slightly hyperactive bowel sounds. The laboratory test results were as follows: white blood count, 2.82 × 10^9^/L; red blood count, 4.07 × 10^12^/L; hemoglobin level, 117 g/L; and platelet count, 102 × 10^9^/L. The liver function test results showed alanine and aspartate aminotransferase levels of 77.9 and 49.2 μ/L, respectively. The erythrocyte sedimentation rate was elevated to 35 mm/h, and the C-reactive protein level was high at 73.7 mg/L. His serum LDH level was 455.9 μ/L. The T-SPOT test result was negative. The laboratory test results were negative for EBV-Ab but positive for EBV-DNA (4.33E + 04 copies/mL). Fungal infection, G, and GM test results were negative. Complete bone marrow aspiration biopsy revealed active proliferation (G = 51.00%, E = 35.00%, and G/E = 1.5/L). The bone marrow and blood cultures were all negative. Intestinal CTE revealed that the lymph nodes in the mesentery and retroperitoneum had increased in number and size. Considering the symptoms of recurring fever and suppurative tonsillitis, the infliximab therapy was suspended, but the symptoms did not respond to any antibiotic or antiviral drugs. Concerning the poor control of the symptoms and the positive test result for EBV-DNA, lymphoma was suspected. Colonoscopy and pathological examinations were repeated. The colonoscopy performed on March 2017 showed that the ileocecal valve was congested, distorted, and remained open. Several huge ulcers were observed in the colon, some of which were circumferential (Fig. 1). Histological examinations revealed several lymphocytes infiltrating the mucosa lamina propria, mucosal base, and vessels. The cells showed mild dysplasia. Acid fast staining was negative. The immunohistochemical results were CK3(+), CD7(+), CD4(+), CD5(60%+), CD8(10%), CD56(−), TiA-1(+), CD21 (FDC net could be seen), CK(+), BCL-2(+), CD30(−), Ki-67(50%+), TDT(−), and CyclinD1(−). In situ hybridisation for EBV-encoded small RNA(EBER) in situ hybridization was > 100/HPF (Fig. 3). Owing to the poor control of the coughing and sore throat, a nasopharyngoscopy was performed. As a soft tissue mass was discovered during nasopharyngoscopy, a biopsy with proper immunohistochemical analysis was performed. Histological examinations revealed an inflammatory background, necrosis, vessel involvement, and neoplastic proliferation of lymphoid cells. The cell nuclei were deformed and atypical. Nucleus fragments were readily apparent. The immunohistochemical results were CD20(10%+), CD79α(−/+), CD45RO(60%+), CD3(+), CD5(+), CD2(80%+), CD56(+), GranzymeB(+), TiA-1(+), and Ki-67(80%+). The EBER in situ hybridization was > 100/HPF (Fig. 4). The biopsies obtained using colonoscopy and nasopharyngoscopy both demonstrated EBV-associated T-cell proliferation disease (level 3, tumor disease). The result of the TCR gene rearrangements was positive. Positron emission tomography-CT revealed soft tissue diffuse thickening and FDG accumulation in the left wall of the nasopharynx (12 × 11 mm, SUVmax = 8.7). The aforementioned results indicated a malignant tumor (lymphoma). FDG accumulated in the posterior wall of the left pharynx and lymph nodes (11 × 10 mm, SUVmax = 5.9) in the bilateral cervical vascular sheath and posterior sternocleidomastoid muscle; thus, lymphoma infiltration was suspected (Fig. 2). This indicated that the lump in the left nasopharynx wall was likely to be cancer (lymphoma). At this point, a diagnosis of EBV-associated T-cell proliferation disease (level 3) was made. The patient was then referred to the hematology department to undergo CHOP treatment but got no good result. The tissue sample from the first colonoscopy was reexamined immunohistochemically. Histological examinations revealed many lymphocytes, plasmocytes, and eosinophils infiltrating the mucosa lamina propria. The crypt was twisted and had branches. The immunohistochemical results were CK(−), CD3(+), CD20(focal+), CD21(follicular net +), and Ki-67(15%+). EBER in situ hybridization was 30/HPF. No obvious sign indicating lymphoma was found in the H&E staining. However, after scrutinizing the pathological section, we found T-cell proliferation. EBER expression was observed as well (Fig. 4). Finally, we correct the former diagnosis as EBV-associated T-cell proliferation disease (level 1).

## 3 Discussion

In this case, the patient initially showed intestinal symptoms. Colonoscopy revealed multiple and multiform ulcers. Several intestinal biopsies indicated inflammation. EBV-associated T-cell proliferation disease was diagnosed only after the patient showed nasopharyngeal symptoms after almost 8 months and after biopsy with proper immunohistochemical analysis using a nasopharyngoscope and another biopsy with colonoscopy was performed. Previously, he was misdiagnosed as having Crohn disease and treated accordingly. The lessons from this case are important.

EBV-associated LPD is usually observed in patients with congenital, acquired, or iatrogenic immunodeficiency, and rarely reported in immunocompetent individuals.^[1,9–12]^ Patients with EBV+T cell LPD show persistent or recurring infectious mononucleosis-like symptoms, including fever, hepatosplenomegaly, lymphadenopathy, liver dysfunction, and high EBV-DNA load in the peripheral blood for at least 3 months.^[1]^ Case reports showed that diarrhea and hematochezia were also common symptoms.^[16,17]^ In this case, the patient was initially immunocompetent. The initial symptom was diarrhea, without fever. He did not have hepatosplenomegaly, lymphadenopathy, and liver dysfunction. These positive symptoms are common in patients with Crohn disease and enterophthisis, but are nonspecific. The clinical features of the present patient were not typical of EBV+T cell LPD. This suggests that clinicians should heighten their vigilance for such cases.

The endoscopy features of the patient were multiple ulcers, which are usually observed in Crohn disease, intestinal tuberculosis (ITB), and lymphoma. Crohn disease is characterized by a discrete and ulcerous transmural inflammation that usually involves the ileocecal region.^[13,14]^ Longitudinal ulcers, aphthous ulcers, cobblestone appearance, luminal stricture, and mucosal bridge favor Crohn disease. Transverse ulcers and a patulous ileocecal valve significantly favor ITB.^[15]^ However, we found that the deep ulcers in this case showed multiform and multifocal features. Xiaodan Zheng reported multiple colorectal ulcers in a patient with EBV-related LPD.^[16]^ Hee Kyong Na reported multiple well-demarcated circumferential or geographic deep ulcers in patients with EBV-related LPD.^[17]^ Moreover, the margin of the ulcer in patients with Crohn disease usually have nodular changes, but no such sign was noticed in the present case. Therefore, in patients with multiform and multiple ulcers with no nodular changes in the margin, the possibility of EBV-associated LPD should be considered.

In terms of pathology, serum EBV-Ab IgM was negative but serum EBV DNA was positive in this case. EBV+T cell LPD is associated with primary EBV infection.^[2]^ EBV Ab in serum seems to have low specificity and cannot be regarded as a standard indicator to rule out EBV infection. EBV DNA quantitative analysis may be a better indicator for detecting EBV infection. EBER detection in tissue is also necessary for diagnosis. At first, this patient was misdiagnosed as Crohn disease, which is difficult to diagnosed because of the rare typical pathological manifestations. However, after the tissue sample from the first colonoscopy was reexamined immunohistochemically carefully, T-cell proliferation and EBER expression were noticed and the right diagnosis was made. Attention should be paid to the clinicopathological results of T-cell proliferation and crypt hyperplasia. In this condition, CD3, TIA-1, and CD56 expressions; EBER; and TCR should be detected. Collaboration between clinicians and pathologists means a lot and may help avoiding midiagnosis.

In this case, we did great in discovering the new symptoms of the patient and correct the diagnosis. However, we failed make a right conclusion at early stage when examining the pathology of the tissue sample from the first colonoscopy. Therefore we conclude that in patients with deep ulcers with multiform and multifocal features, the possibility of not only Crohn disease, ITB, and lymphoma but also EBV-related intestinal disease such as EBV-associated LPD should be considered. EBV-associated LPD shows a fulminant clinical course that results in high mortality rates.^[1]^ Therefore, early diagnosis is clearly significant. From this case, we learned that some indicators are helpful to improve the diagnosis rate of EBV-associated LPD, such as multiform and multifocal deep ulcers, repeated multiple biopsy, gene rearrangement, EBV DNA quantitative analysis result, and EBER.

## Author contributions

**Data curation:** Dalian Ou, Chunhui Ouyang.

**Writing – original draft:** Sizhu Wang.

**Writing – review & editing:** Yinghuan Dai, Jie Zhang, Fanggen Lu.

Sizhu Wang orcid: 0000-0002-4509-7550.
